# A Study on Chemical Characterization and Biological Abilities of *Alstonia boonei* Extracts Obtained by Different Techniques

**DOI:** 10.3390/antiox11112171

**Published:** 2022-11-01

**Authors:** Adriano Mollica, Gokhan Zengin, Kouadio Ibrahime Sinan, Marcella Marletta, Stefano Pieretti, Azzurra Stefanucci, Ouattara Katinan Etienne, József Jekő, Zoltán Cziáky, Mir Babak Bahadori, Carene Picot-Allain, Mohamad Fawzi Mahomoodally

**Affiliations:** 1 Department of Pharmacy, University “G. d’Annunzio” of Chieti-Pescara, 66100 Chieti, Italy; 2 Department of Biology, Faculty of Science, Selcuk University, Campus, 42250 Konya, Turkey; 3 University Guglielmo Marconi, 00193 Rome, Italy; 4 National Centre for Drug Research and Evaluation, Istituto Superiore di Sanità, 00161 Rome, Italy; 5 Laboratoire de Botanique, UFR Biosciences, Université Félix Houphouët-Boigny, Abidjan 83111, Côte d’Ivoire; 6 Agricultural and Molecular Research and Service Institute, University of Nyíregyháza, 4400 Nyíregyháza, Hungary; 7 Medicinal Plants Research Center, Maragheh University of Medical Sciences, Maragheh 83111, Iran; 8 Department of Health Sciences, Faculty of Medicine and Health Sciences, University of Mauritius, Réduit 230, Mauritius; 9 Center for Transdisciplinary Research, Department of Pharmacology, Saveetha Dental College, Saveetha Institute of Medical and Technical Science, Chennai 600077, India; 10 Centre of Excellence for Pharmaceutical Sciences, North-West University, Private Bag X6001, Potchefstroom 2520, South Africa

**Keywords:** *Alstonia*, phenolics, extraction methods, anti-inflammatory, natural agents

## Abstract

In the quest for novel therapeutic agents from plants, the choice of extraction solvent and technique plays a key role. In this study, the possible differences in the phytochemical profile and bioactivity (antioxidant and enzyme inhibitory activity) of the *Alstonia boonei* leaves and stem bark extracted using water, ethyl acetate and methanol, and different techniques, namely infusion, maceration and Soxhlet extraction, were investigated. Data collected showed that methanol extracts of both *A. boonei* leaves (48.34–53.08 mg gallic acid equivalent [GAE]/g dry extract) and stem bark (37.08–45.72 mg GAE/g dry extract) possessed higher phenolic content compared to the ethyl acetate extracts (leaves: 30.64–40.19 mg GAE/g; stem bark: 34.25–35.64 mg GAE/g). The methanol extracts of *A. boonei* leaves showed higher radical scavenging and reducing capacity, and these findings were in accordance with phenolic content results. In general, water extracts of *A. boonei* leaves and stem bark obtained by infusion were poor inhibitors of acetylcholinesterase, α-amylase, α-glucosidase, and tyrosinase, except for butyrylcholinesterase. The chemical profiles of the extracts were determined by UHPLC–MS and the presence of several compounds, such as phenolic acids (caffeic, chlorogenic and ferulic acids, etc.), flavonoids (rutin and isoquercetin) and flavonolignans (Cinchonain isomers). Cell viability was tested using the human peripheral blood monocytic cell line (THP-1), and the extracts were safe up to 25 μg/mL. In addition, anti-inflammatory effects were investigated with the releasing of IL-6 TNF-α and IL-1β. In particular, stem bark extracts exhibited significant anti-inflammatory effects. Data presented in this study highlight the key role of solvent choice in the extraction of bioactive secondary metabolites from plants. In addition, this study appraises the antioxidant and enzyme inhibitory action of *A. boonei* leaves and stem bark, which are extensively used in traditional medicine.

## 1. Introduction

*Alstonia boonei*, belonging to the Apocynaceae family, has been extensively used in traditional medicine. This ethnomedicinal plant, commonly found in tropical and subtropical Africa, Australia, Southeast Asia, and Central America, was found to exhibit several biological and pharmacological actions [1]. *A. boonei* is a large deciduous tree, measuring up to 45 m, with a deeply fluted trunk that can reach 1.2 m in diameter and a greyish-green or grey bark, from which, a copious milky latex is exuded [2]. In ethnomedicine, *A. boonei* is used to treat malaria, sore throat, cough, toothache, snake bites, ulcer, jaundice, skin conditions, arthritis, rheumatism and hypertension, and is also used as antihelminthic [1,2,3]. Pharmacological studies conducted on *A. boonei* stem bark methanol extract established its anti-inflammatory, analgesic and antipyretic activities [4]. *A. boonei* combined with *Khaya ivorensis* exhibited antiplasmodial activity in the murine malaria model, thereby validating its traditional use in the treatment of malaria [5]. Traditional use of *A. boonei* as an anti-inflammatory agent was validated by a study conducted by Enechi, Odo and Onyekwelu [2], who reported that the ethanol extract of the stem bark of *A. boonei* exhibited a remarkable inhibitory effect on leucocyte migration. An aqueous fraction of 70% methanol extract of *A. boonei* leaves demonstrated significant anti-inflammatory and antioxidant activities in carrageenan and formaldehyde-induced arthritic rats [1]. *A. boonei* stem bark ethanol extract showed inhibitory action against *Escherichia coli* [6]. The ethyl acetate extract of *A. boonei* leaves showed potent inhibitory activity against key enzymes targeted in the management of diabetes type II, namely, α-amylase (IC_50_: 3.17 mg/mL) and α-glucosidase (IC_50_: 0.70 mg/mL). Besides, administration of ethyl acetate extract to starch-loaded Wistar rats showed a significant reduction in the blood glucose level of the rats within 2 h [7].

The plant secondary metabolites present possess versatile therapeutic actions and have been shown to exhibit inhibitory action on several enzymes. In this sense, plant secondary metabolites capable of mitigating the activity of enzymes targeted in the management of diabetes type II, Alzheimer’s disease and epidermal hyperpigmentation represent interesting possibilities for new drug development. Diabetes type II, the most common type of diabetes, is a chronic metabolic condition, which is characterised by hyperglycemia as a result of defective insulin secretion or functioning [8]. Alzheimer’s disease is the most common neurodegenerative geriatric condition, characterized by progressive memory impairment and cognitive deficits [9]. The incidence and prevalence of diabetes type II and Alzheimer’s disease are rapidly growing, and are affecting millions of individuals globally. It has been claimed that anti-diabetic agents possessing low or no inhibitory action against α-amylase were favorable, since high α-amylase inhibition has been associated with poor digestion of ingested carbohydrates, causing abdominal discomfort [10]. In this sense, one of the therapeutic strategies used to manage diabetes type II focuses on the inhibition of α-glucosidase, which is situated in the epithelial mucosa of the small intestine [11]. Tyrosinase, an enzyme containing copper, is essential for the biosynthesis of melanin, a brown pigment that shields human skin from ultraviolet radiation [12]. However, epidermal hyperpigmentation problems and dermatological conditions are more likely to develop when melanin production and accumulation are excessive. Cosmetic and dermatological tyrosinase inhibitors are used to treat hyperpigmented skin conditions such as acne scars, age spots and melasma [13]. 

In the quest for novel therapeutic agents from natural sources, namely plants, extraction is a fundamental step that will determine the phytochemical profile of the extracts, and subsequently, their bioactivity. In fact, the choice of the extraction solvent and technique has always been a challenge for researchers. Several studies have shown the significant difference in bioactivity of plant extracts prepared from different solvents. Indeed, the choice of the extraction solvent depends on the nature of phytochemicals being targeted, in case the compound is known. However, in the quest for novel bioactive compounds with unknown structures, extracting using solvents of different polarities might provide better insight into the phytochemical profile of a plant, and eventually help identify interesting bioactive compounds. 

In this regard, the present study set out to investigate the possible differences in the phytochemical profile and bioactivity (antioxidant, enzyme inhibitory and anti-inflammatory activity) of the *A. boonei* leaves and stem bark extracted using different solvents, namely water, ethyl acetate and methanol, and using different extraction techniques, namely, infusion, maceration and Soxhlet extraction. The chemical compounds of the extracts were characterized by the UHPLC–MS technique. It is expected that data gathered from the present investigation will provide an insight into the possible effects of extraction solvents and methods on the observed bioactivity of *A. boonei* leaves and stem bark. The obtained results could open a new horizon in the production of functional applications with *A. boonei* leaves or stem bark.

## 2. Materials and Methods

### 2.1. Plant Material and Preparation of Extracts

In the summer of 2019, the leaves and stem bark of *A. boonei* were harvested in the village of Prikro (Brobo City, Côte d’Ivoire). The National Floristic Centre (The Université Félix Houphout-Boigny, Abidjan, Côte d’Ivoire) identified the plant. Deposits of voucher specimens were made at the herbarium of the aforementioned institution. Leaves and stem barks were carefully separated, and they were dried under dark conditions for one week at room temperature.

Ethyl acetate, methanol and water were used as solvents in the present study. Methanol allows for the extraction of both hydrophilic and hydrophobic compounds from plant materials, and thus, we could gain more insights for plant extracts. It has been shown in the literature that methanol is commonly used and is more effective as an extraction solvent for *Alstonia boonei* [14,15,16,17,18,19]. With this in mind, methanol was selected as one solvent for the current study. 

In the preparation of plant extracts, we used three techniques: maceration, Soxhlet and infusion. The maceration technique was performed either stirred or not stirred. Using the technique, the plant materials (10 g) were stirred with 200 mL of the solvents (ethyl acetate or methanol) at 250 rpm for 24 h at room temperature. Without stirring, the plant materials (10 g) were kept in the solvents (ethyl acetate or methanol) for 24 h in the dark at room temperature. With the Soxhlet method, the plant materials (10 g) were extracted with the solvents (ethyl acetate or methanol) in a Soxhlet apparatus for 6 h. After the duration of extraction, each extract was filtered with Whatman filter paper and the solvents were removed with a rotary-evaporator. In infusion, the plant material (10 g) was steeped in boiling water (200 mL) for 15 min before being filtered. For 48 h, the mixture was lyophilized to remove water. All extracts were kept at 4 °C until analysis. 

### 2.2. Profile of Bioactive Compounds

The extracts were dissolved in methanol (for ethyl acetate and methanol extracts) and water (for infusion). Quantification of the total phenolic and flavonoid content was performed using Folin–Ciocalteu and AlCl3 assays, respectively [20]. Gallic acid equivalents (mg GAEs/g extract) and rutin equivalents (mg REs/g extract) were used to describe the outcomes of the two tests. All experimental details are given in the Supplemental Materials.

### 2.3. UHPLC–MS Analysis

Compositions of the different extracts were determined using a Dionex Ultimate 3000RS UHPLC instrument(Thermo Scientific, Waltham, MA, USA). The extract was filtered through a 0.22 μm PTFE filter membrane (Labex Ltd., Budapest, Hungary) before HPLC analysis. Extracts were injected onto a Thermo Accucore C18 (100 mm × 2.1, mm i. d., 2.6 μm) column themostated at 25 °C (±1 °C). The solvents used were water (A) and methanol (B). Both were acidified with 0.1 % formic acid. The flow rate was maintained at 0.2 mL min^−1^. The elution gradient was isocratic 5 % B (0–3 min), a linear gradient increasing from 5% B to 100% (3–43 min), 100% B (43–61 min), a linear gradient decreasing from 100% B to 5% (61–62 min) and 5 % B (62–70 min). The column was coupled with a Thermo Q-Exactive Orbitrap mass spectrometer (Thermo Scientific, Waltham, MA, USA) equipped with electrospray ionization source. Spectra were recorded in positive- and negative-ion mode [21].

### 2.4. Determination of Antioxidant and Enzyme Inhibitory Effects

For antioxidant and enzyme inhibitory assays, the extracts were dissolved in methanol (for ethyl acetate and methanol extracts) and water (for infusion). Methods for measuring the extracts’ antioxidant and enzyme-inhibiting properties were previously described [22,23]. Trolox (for radical scavenging and reducing power assays) and EDTA were used as positive controls. Each of the radical scavenging activities (ABTS^•+^, DPPH^•^), as well as the reducing capacities (CUPRAC and FRAP), were reported in milligrams of Trolox equivalent (TE) per milligram of extract. Total antioxidant activity (phosphomolybdenum assay, PBD) was reported in mmol TE/g extract, while metal chelating ability (MCA) was reported as mg EDTAE/g extract. The inhibitory activities were tested against AChE, BChE, tyrosinase, amylase and glucosidase. Galanthamine (for AChE and BChE), kojic acid (for tyrosinase) and acarbose (for amylase and glucosidase) were used as standard enzyme inhibitors. The results were expressed as the equivalents of the standards (galanthamine equivalents (GALAE), kojic acid equivalents (KAE) and acarbose equivalents (ACAE). All experimental details are given in the Supplemental Materials.

### 2.5. Cell Line

THP-1 cells, which originate from human peripheral blood mononuclear cells, were obtained from the American Type Culture Collection (Bethesda, MD, USA). Cells were grown in a 37 °C, 5% CO_2_ incubator with RPMI-1640 medium containing 2 mM L-glutamine, 100 U/mL streptomycin-penicillin and 10% heat-inactivated fetal bovine serum (Sigma Aldrich, St. Louis, MO, USA). At 37 degrees Celsius for 24 h, 1 × 10^6^ THP-1 cells were plated in 6-well culture plates and differentiated into macrophages with 100 ng/mL phorbol-12-myristate-13-acetate (PMA, St. Louis, MO, USA).

### 2.6. MTT Assay

Once THP-1 monocytes had been differentiated into macrophages, the effect of *A. boonei* extracts on the viability of the LPS-stimulated macrophages was determined using the 3-(4,5-dimethylthiazol-2-yl)-2,5-diphenyltetrazolium bromide (MTT) assay. The extracts were solubilized in RPMI medium containing 0.1% DMSO. Twenty microliters of MTT (1 mg/mL in PBS) were added to each well after *A. boonei* had been present for 24 h at 25, 50 and 100 μg/mL, and the plates were incubated for 4 h under standard culture conditions. After that, 100 μL of DMSO was added to the cells. With the help of a microplate reader (MultiskanTM FC Microplate Photometer, Thermo Scientific^TM^, Waltham, MA, USA), the absorbance was determined to be 570 nm. The data were expressed as a percentage, with 100% corresponding to the value obtained for the solvent control.

### 2.7. IL-6, TNF-α and IL-1β Assays for Anti-Inflammatory Activity

In order to increase cytokine production, macrophages were treated with LPS at a final concentration of 0.1 μg/mL, and then with *A. boonei* extracts at 25, 50 and 100 μg/mL. The extracts were solubilized in RPMI medium containing 0.1% DMSO. Dexamethasone (Sigma Aldrich, USA) was employed as a positive control (0.04 μg/mL). A centrifuge was used to separate the supernatant from the cells after 24 h of incubation. Quantification of IL-6, IL-1β and TNF-α secretion was achieved by following the ELISA manufacturer’s instructions (R&D Systems, Minneapolis, MN, USA). The ELISA results were normalized to the MTT values to cut down on the variation that could have resulted from differences in cell density. Cytokine concentration in the negative control (cells treated with LPS alone) was set at 100%. All data from the *A. boonei* extract tests were normalized by dividing them by the value obtained from the negative control.

### 2.8. Statistical Analysis

The results of three independent experiments were used to calculate the values for antioxidant and enzyme inhibitory assays (mean ± SD). One-way ANOVA with Tukey’s assay was used to compare the extracts’ antioxidant and enzyme-inhibiting properties. To conduct the statistical analysis, XlStat 16.0 (Addinsoft Inc., New York, NY, USA)was used. 

The values are typically presented in a mean ±SEM format in cellular analysis. One-way analysis of variance was used to determine statistical significance between means, and Dunnett’s multiple comparisons test was used to further examine the data. The significance level of 0.05 was considered to be statistically significant. For the statistical analysis, we used GraphPad Prism 9.0 (San Diego, CA, USA).

## 3. Results and Discussion

### 3.1. Total Phenolic and Flavonoid Content

The total phenolic and flavonoid contents of the different extracts of *A. boonei* leaves and stem bark are summarised in Table 1. In general, methanol extracts showed higher phenolic contents as compared to the ethyl acetate extracts, suggesting that methanol was a better extracting solvent as compared to ethyl acetate. It was also observed that *A. boonei* leaves contained higher flavonoid content compared to the stem bark (Table 1). It is worth mentioning that the flavonoid content of ethyl acetate extracts of *A. boonei* stem bark was higher compared to the methanol extracts. The water extracts obtained by infusion also showed high phenolic content. Alkaloids, tannins, saponins, flavonoids, cardiac glycosides and ascorbic acid were previously identified in the methanol and water extracts *A. boonei* stem bark [14].

### 3.2. Chemical Characterization

UHPLC–MS analysis led to the characterization of plant metabolites in the extracts of *A. boonei*. Obtained data, including identity of compounds, their molecular formula, mass fragments and retention times can be found in Table 2, Table 3, Table 4, Table 5, Table 6 and Table 7. Total ion chromatograms are given in Supplemental Materials (Figures S1–S12). Some of the characterized metabolites are well-known bioactive compounds, such as chlorogenic acid, caffeic acid, 4-coumaric acid and quercetin. These phenolic compounds have strong bioactivities, including antioxidant, antimicrobial, anti-inflammatory, neuroprotective, hypotensive and cardioprotective effects [24,25,26,27]. Quercetin derivatives, such as rutin, quercitrin and isoquercitrin, were other common compounds in the investigated extracts. These are important natural products exerting valuable therapeutic effects [28,29]. Most of the observed antioxidant effects; reducing ability, radical scavenging, enzyme inhibitory and anti-inflammatory activities, from different extracts of *A. boonei* could be related to these phenolic and flavonoid glycosides. In addition to the mentioned compounds, other natural substances, such as loganic acid (an iridoid), voacangine (an alkaloid) and quinic acid (a cyclitol), were also found in *A. boonei*. The highest number of compounds was found in Mac-MeOH- not stirred (61), and the lowest number of compounds was identified in Mac-ET- not stirred (26). To the best of our knowledge, this work is the first comprehensive phytochemical analysis on different parts and extracts of *A. boonei*.

### 3.3. Antioxidant Effect

A comprehensive study of the antioxidant activity of the *A. boonei* leaves and stem bark extracts obtained from infusion, maceration and Soxhlet extraction using water, ethyl acetate and methanol was carried out. Results of the antioxidant activities determined by ABTS^•+^, DPPH^•^, CUPRAC, FRAP and metal chelating are shown in Table 8. In line with the total phenolic results, the antioxidant activity of the leaves extracts was higher compared to the stem bark extracts, and higher activity was observed for the methanol extracts. The ability of the extracts to scavenge free radicals, namely, ABTS^•+^ and DPPH^•^, was determined. The water extract of *A. boonei* leaves obtained by infusion showed the highest ABTS^•+^ and DPPH^•^ scavenging ability. It was also observed that the leaves extracts were more potent radical scavengers compared to the stem bark extracts. The presence of antioxidant compounds, such as phenolics and phenolic acids, causes the TPTZ-Fe^3+^ complex to be reduced to the TPTZ-Fe^2+^ complex, yielding a chromophore with maximum absorption at 593 nm, and the neocuproine-Cu^2+^ complex to be reduced to the neocuproine-Cu^+^ complex, yielding an orange–yellow chromophore with maximum absorption at 450 nm. The methanol extract of *A. boonei* leaves showed the highest reducing activity. The metal chelating potential of *A. boonei* leaves and stem bark extracts was evaluated and reported in Table 8. The water extract of the stem bark showed the highest chelating ability while none of the ethyl acetate extracts of *A. boonei* stem bark were active.

### 3.4. Enzyme Inhibitory Effects

In the present study, the inhibitory action of *A. boonei* leaves and stem bark extracts on cholinesterase enzymes, namely, acetylcholinesterase (AChE) and butyrylcholinesterase (BChE), targeted in the management of neurodegenerative diseases, such as Alzheimer’s disease, was studied and reported in Table 9. Both *A. boonei* leaves and stem bark water extracts obtained by infusion showed no activity against AChE, while inhibition was noted against BChE. It was also observed that the value for AChE inhibition ranged from 4.89–5.57 mg GALAE/g for *A. boonei* leaves extracts and 4.91–5.78 mg GALAE/g for stem bark extracts, showing no significant variations among the different extracts. On the other hand, variable inhibitory action was observed against BChE (Table 9). It is worth highlighting that water extract of *A. boonei* stem bark showed the highest inhibitory activity against BChE. In general, *A. boonei* leaves and stem bark extracts showed low inhibitory activity against α-amylase. In Table 9, it is noted that the ethyl acetate extracts of *A. boonei* leaves and both water extracts showed no inhibitory action against α-glucosidase. However, ethyl acetate and methanol extracts of *A. boonei* stem bark inhibited α-glucosidase. The ability of *A. boonei* leaves and stem bark extracts to inhibit tyrosinase activity was also assessed and presented in Table 9. Ethyl acetate and methanol extracts of both *A. boonei* leaves and stem bark were active inhibitors of tyrosinase. In accordance with total phenolic results, ethyl acetate extracts showed lower inhibitory action against tyrosinase compared to their corresponding methanol extracts. Poor inhibition was observed for the water extracts. 

### 3.5. Cell Viability

The cell viability percentages can be verified by the MTT (Table 10) in the groups treated at different concentrations of *Alstonia boonei* extracts (25, 50 and 100 µg/mL) and the control group. *Alstonia boonei* extracts did not induce significant cell viability reduction at the concentration of 25 µg/mL. A decrease of the cell viability percentage of macrophage at 50 and 100 µg/mL was observed after exposure to the ethyl acetate extracts. A reduction of cell viability was observed at 100 µg/mL, but not at 50 µg/mL, after exposure to the methanol extracts. There was no significant cell viability reduction after macrophage exposure to infusions. The results indicated that all the extracts were safe up to 25 μg/mL to conduct the assay of anti-inflammatory activity.

### 3.6. Anti-Inflammatory Activity

The levels of IL-6, TNF- and IL-1 in macrophage culture supernatants were measured using an ELISA kit, and then the anti-inflammatory effects of *Alstonia boonei* extracts on LPS-stimulated macrophages were studied. LPS-induced macrophages were shown to have significantly increased production of pro-inflammatory cytokines, while dexamethasone reduced it (Table 10). After cell exposition to leaves—infusion, the results demonstrated that IL-6, IL-1β and TNF-α production was significantly downregulated in LPS-induced macrophages treated with the extracts at the highest concentrations of 50 and 100 µg/mL (Table 10). On the contrary, the ethyl acetate extract of leaves from maceration reduced cytokine release induced by LPS in macrophages at the concentration of 100 µg/mL only (Table 10). Cells treated with the methanol extract of leaves from maceration showed a reducing effect on IL-6, TNF-α and IL-1β production at 50 and 100 µg/mL (Table 10). The stem bark—infusion and maceration—methanol extracts appear to be the most effective of the series in reducing the release of pro-inflammatory cytokines, since at all the concentrations used (Table 10). The ethyl acetate extract of stem bark reduced cytokine release at 100 µg/mL, whereas no effects were observed at the concentration of 10 and 50 µg/mL (Table 10).

## 4. Conclusions

This study provided scientific evidence that *A. boonei* leaves and stem bark have biological activities, specifically, antioxidant and enzyme inhibitory properties. In the same extraction methods, namely, maceration and Soxhlet, the solvents were affected to chemical composition and biological activities. Generally, the methanol extracts for both parts exhibited more antioxidant abilities when compared to ethyl acetate and water extracts. Based on the parts, the extracts of leaves were more active in the antioxidant assays. The ethyl acetate and methanol showed greater AChE, tyrosinase and amylase inhibitory effects than did infusions. In addition, the chemical composition of the extracts depended on the extraction solvents, and the methanol extracts contained more components compared to ethyl acetate and water extracts. In the UHLPC–MS analysis, the presence of bioactive compounds, such as quinic acid, caffeic acid, rutin and isoquercetin, was found. In particular, stem bark extracts showed great anti-inflammatory potential. From the results, methanol could be useful for the preparation of further applications using *A. booeni* at industrial scale. However, the toxic properties of methanol should not be forgotten, and ethanol could be used in these applications. Future experiments, including animal and bioavailability studies, should be conducted to corroborate the findings.

## Figures and Tables

**Table 1:** Total bioactive compounds and total antioxidant capacity (by phosphomolybdenum assay) of the studied extracts *.

Parts	Extraction Methods/Solvent	**Total Phenolic Content** **(mg GAE/g)**	**Total Flavonoid Content** **(mg RE/g)**	**Phosphomolybdenum** **(mmol TE/g)**
Leaves	Infusion-water	51.08 ± 0.21 ^b^	4.18 ± 0.16 ^e^	2.15 ± 0.08 ^cd^
	MAC-EA	30.64 ± 0.38 ^f^	34.51 ± 0.39 ^c^	1.78 ± 0.04 ^d^
	MAC-MeOH	48.34 ± 0.38 ^c^	37.28 ± 0.56 ^b^	2.55 ± 0.27 ^abc^
	MAC-EA (not stirred)	35.81 ± 0.50 ^e^	34.81 ± 0.46 ^c^	2.61 ± 0.15 ^ab^
	MAC-MeOH (not stirred)	53.08 ± 0.24 ^a^	37.65 ± 0.20 ^b^	2.78 ± 0.15 ^a^
	SE-EA	40.19 ± 0.67 ^d^	8.14 ± 0.31 ^d^	2.25 ± 0.09 ^bc^
	SE-MeOH	49.27 ± 0.30 ^c^	39.10 ± 0.42 ^a^	2.37 ± 0.13 ^bc^
Stem bark	Infusion-water	31.99 ± 0.15 ^f^	0.54 ± 0.08 ^e^	1.30 ± 0.02 ^c^
	MAC-EA	35.64 ± 0.62 ^d^	3.05 ± 0.09 ^b^	2.06 ± 0.04 ^a^
	MAC-MeOH	39.64 ± 0.03 ^b^	1.85 ± 0.06 ^d^	1.54 ± 0.08 ^bc^
	MAC-EA (not stirred)	34.38 ± 0.44 ^de^	2.69 ± 0.08 ^bc^	1.84 ± 0.13 ^ab^
	MAC- MeOH (not stirred)	45.72 ± 0.28 ^a^	2.45 ± 0.08 ^c^	1.91 ± 0.22 ^a^
	SE-EA	34.25 ± 0.42 ^e^	4.07 ± 0.33 ^a^	1.60 ± 0.09 ^bc^
	SE-MeOH	37.08 ± 0.79 ^c^	2.76 ± 0.06 ^bc^	1.54 ± 0.07 ^bc^

* Values expressed are means ± S.D. of three parallel measurements. GAE: Gallic acid equivalent; RE: Rutin equivalent; TE: Trolox equivalent. MAC: Maceration; SE: Soxhlet extraction; EA: Ethyl acetate; MeOH: Methanol. Different letters indicate significant differences in the tested extracts of each parts (*p* < 0.05).

**Table 2:** Chemical characterization of leaves—infusion.

No.	Name	Formula	Rt	[M + H]^+^	[M − H]^−^	Fragment 1	Fragment 2	Fragment 3	Fragment 4	Fragment 5	Literature
1	Unidentified dihydroxybenzoic acid derivative	C_22_H_20_O_12_	13.58		475.08765	299.0776	153.0181	137.0232	109.0281		
2 ^1^	Chlorogenic acid (3-O-Caffeoylquinic acid)	C_16_H_18_O_9_	14.86	355.10291		163.0390	145.0285	135.0442	117.0338	89.0388	
3	3-O-Feruloylquinic acid	C_17_H_20_O_9_	15.11		367.10291	193.0500	191.0552	173.0443	134.0361	93.0333	
4	Caffeic acid	C_9_H_8_O_4_	15.16		179.03444	135.0439	107.0487				
5	Loganic acid	C_16_H_24_O_10_	15.72		375.12913	213.0764	169.0860	151.0751	113.0230	69.0330	
6	Vallesamine or isomer	C_20_H_24_N_2_O_3_	15.79	341.18652		309.1599	236.1062	208.1128	194.0967	168.0807	
7	Chryptochlorogenic acid (4-O-Caffeoylquinic acid)	C_16_H_18_O_9_	16.21	355.10291		163.0390	145.0285	135.0442	117.0339	89.0390	
8	4-O-(4-Coumaroyl)quinic acid cis isomer	C_16_H_18_O_8_	16.27		337.09235	191.0557	173.0445	163.0389	119.0488	93.0330	
9	Swertiamarin or isomer	C_16_H_22_O_10_	16.73	375.12912		213.0758	195.0655	177.0549	151.0391	107.0496	
10	Lochnericine or isomer	C_21_H_24_N_2_O_3_	17.02	353.18652		335.1773	291.1494	166.0863	158.0966	144.0808	
11	Unidentified N-formylalkaloid 1 isomer 1	C_22_H_24_N_2_O_5_	17.30	397.17635		369.1812	337.1547	299.1392	267.1128	224.0704	
12	Secologanoside	C_16_H_22_O_11_	17.32		389.10839	345.1197	209.0451	183.0657	165.0545	69.0330	
13	Lochnericine or isomer	C_21_H_24_N_2_O_3_	17.39	353.18652		335.1745	291.1493	166.0864	158.0966	144.0808	
14	5-O-(4-Coumaroyl)quinic acid	C_16_H_18_O_8_	17.51		337.09235	191.0554	173.0445	163.0389	119.0488	93.0331	
15	Picralinal or isomer	C_21_H_22_N_2_O_4_	17.65	367.16578		349.1910	339.1704	307.1441	269.1283	180.1020	
16	Quercetin-O-hexoside-O-rutinoside isomer 1	C_33_H_40_O_21_	17.71		771.19839	609.1485	462.0805	301.0355	300.0285	299.0202	
17 ^2^	Echitamine	C_22_H_29_N_2_O_4_	17.92	385.21273		367.2011	349.1919	310.1438	250.1227	220.1121	[30]
18	Quercetin-O-hexoside-O-rutinoside isomer 2	C_33_H_40_O_21_	17.96		771.19839	609.1474	463.0885	462.0807	301.0354	299.0201	
19	Sweroside or isomer	C_16_H_22_O_9_	18.03	359.13421		197.0811	179.0704	151.0754	127.0392	111.0809	
20	Picralinal or isomer	C_21_H_22_N_2_O_4_	18.04	367.16578		349.1948	339.1704	307.1442	269.1285	194.0603	
21	Quebrachidine or isomer	C_21_H_24_N_2_O_3_	18.05	353.18652		335.1773	324.1595	303.1491	293.1651	275.1539	
22	Unidentified N-formylalkaloid 1 isomer 2	C_22_H_24_N_2_O_5_	18.11	397.17635		369.1813	337.1546	299.1393	256.0970	224.0709	
23	4-O-(4-Coumaroyl)quinic acid	C_16_H_18_O_8_	18.13		337.09235	191.0556	173.0445	163.0390	119.0489	93.0331	
24	Nα-Formyl-12-methoxyechitamidine	C_22_H_26_N_2_O_5_	18.14	399.19200		371.1967	339.1705	299.1406	267.1127	198.0913	[31]
25 ^1^	4-Coumaric acid	C_9_H_8_O_3_	18.50		163.03952	119.0488	93.0331				
26	5-O-Feruloylquinic acid	C_17_H_20_O_9_	18.51		367.10291	193.0501	191.0553	173.0445	134.0361	93.0330	
27	Vallesamine or isomer	C_20_H_24_N_2_O_3_	18.55	341.18652		309.1598	237.1023	209.1074	194.0960	168.0806	
28	N-Formylechitamidine	C_21_H_24_N_2_O_4_	18.91	369.18143		351.1710	341.1862	309.1599	202.0874		[32]
29	4-O-Feruloylquinic acid	C_17_H_20_O_9_	19.03		367.10291	193.0501	191.0554	173.0445	134.0362	93.0330	
30	5-O-(4-Coumaroyl)quinic acid cis isomer	C_16_H_18_O_8_	19.70		337.09235	191.0554	173.0444	163.0389	119.0487	93.0331	
31	Unidentified N-formylalkaloid 2	C_22_H_24_N_2_O_4_	20.33	381.18143		353.1862	339.1706	321.1599	263.1175	212.0942	
32	Secologanoside methyl ester	C_17_H_24_O_11_	20.76		403.12404	371.0987	223.0606	179.0551	165.0545	121.0281	
33	Lagumicine or isomer	C_20_H_22_N_2_O_4_	21.06	355.16578		337.1548	214.0863	200.0709	182.0603	154.0652	
34	Akuammicine	C_20_H_22_N_2_O_2_	21.47	323.17596		294.1491	291.1495	280.1342	263.1543	234.1286	
35	Unidentified alkaloid isomer 1	C_21_H_24_N_2_O_4_	21.55	369.18143		337.1545	309.1619	299.1390	267.1128	224.0703	
36	Echitamidine	C_20_H_24_N_2_O_3_	21.87	341.18652		323.1757	309.1581	202.0863	142.0653	140.1072	[30]
37	N-Methylakuammicine	C_21_H_24_N_2_O_2_	22.03	337.19161		309.1607	305.1650	294.1504	277.1700	263.1543	
38	Unidentified alkaloid isomer 2	C_21_H_24_N_2_O_4_	22.06	369.18143		337.1547	309.1598	299.1392	256.0967	224.0708	
39	Akuammicine isomer N-methyl derivative	C_21_H_24_N_2_O_2_	22.50	337.19161		305.1649	277.1700	263.1543	248.1086	222.1277	
40	Akuammicine isomer 1	C_20_H_22_N_2_O_2_	22.70	323.17596		291.1494	280.1332	263.1549	249.1385	234.1279	
41	Tubotaiwine	C_20_H_24_N_2_O_2_	22.88	325.19160		293.1648	265.1335	236.1440	222.1286	194.0960	
42	Akuammicine isomer 2	C_20_H_22_N_2_O_2_	23.11	323.17596		291.1494	280.1335	263.1528	249.1387	234.1285	
43	17-O-Acetyl-N-demethylechitamine or isomer	C_23_H_28_N_2_O_5_	23.16	413.20765		395.1964	353.1862	335.1760	292.1334	232.1123	
44 ^1^	Isoquercitrin (Quercetin-3-O-glucoside)	C_21_H_20_O_12_	23.48		463.08765	301.0357	300.0280	271.0251	255.0300	151.0022	
45 ^1^	Rutin (Quercetin-3-O-rutinoside)	C_27_H_30_O_16_	23.56		609.14557	301.0357	300.0278	271.0251	255.0298	151.0024	
46	Voacangine	C_22_H_28_N_2_O_3_	24.77	369.21782		337.1911	309.1609	266.1160	252.1022		[33]
47	Akuammidine	C_21_H_24_N_2_O_3_	24.95	353.18652		321.1599	310.1412	293.1650	264.1369	250.1236	[33]
48 ^1^	Quercitrin (Quercetin-3-O-rhamnoside)	C_21_H_20_O_11_	25.03		447.09274	301.0357	300.0280	271.0252	255.0299	151.0025	
49	Dihydroakuammidine or isomer	C_21_H_26_N_2_O_3_	25.84	355.20217		323.1756	295.1441	266.1541	252.1388	224.1061	
50 ^1^	Quercetin (3,3′,4′,5,7-Pentahydroxyflavone)	C_15_H_10_O_7_	27.58		301.03484	273.0423	178.9975	151.0026	121.0283	107.0124	
51	9-Hydroxyoctadecatrienoic acid	C_18_H_30_O_3_	40.17		293.21167	275.2022	231.2111	171.1017	121.1009	59.0132	
52	13-Hydroxyoctadecatrienoic acid	C_18_H_30_O_3_	40.31		293.21167	275.2023	235.1703	223.1335	195.1386	179.1433	
53	Linoleamide	C_18_H_33_NO	44.49	280.26404		263.2341	245.2264	109.1016	95.0861	81.0705	
54	Oleamide	C_18_H_35_NO	45.75	282.27969		265.2526	247.2421	135.1172	83.0861	69.0705	

^1^ Confirmed by standard. ^2^ [M]^+^.

**Table 3:** Chemical characterization of leaves—MAC-EA (not stirred).

No.	Name	Formula	Rt	[M + H]^+^	[M − H]^−^	Fragment 1	Fragment 2	Fragment 3	Fragment 4	Fragment 5	Literature
1	Quinic acid	C_7_H_12_O_6_	1.28		191.05557	173.0444	171.0286	127.0387	111.0437	93.0330	
2 ^1^	Chlorogenic acid (3-O-Caffeoylquinic acid)	C_16_H_18_O_9_	14.95	355.10291		163.0390	145.0285	135.0443	117.0338	89.0389	
3	Caffeic acid	C_9_H_8_O_4_	15.18		179.03444	135.0439	107.0483				
4	Loganic acid	C_16_H_24_O_10_	15.75		375.12913	213.0764	169.0857	151.0755	113.0230	69.0330	
5	Swertiamarin or isomer	C_16_H_22_O_10_	16.78	375.12912		213.0757	195.0655	177.0547	151.0390	107.0495	
6	Secologanoside	C_16_H_22_O_11_	17.32		389.10839	345.1188	209.0453	183.0652	165.0547	69.0330	
7	Unidentified N-formylalkaloid 1 isomer 1	C_22_H_24_N_2_O_5_	17.42	397.17635		369.1809	337.1545	299.1388	267.1129	224.0708	
8	5-O-(4-Coumaroyl)quinic acid	C_16_H_18_O_8_	17.44		337.09235	191.0555	173.0447	163.0388	119.0486	93.0331	
9	Lochnericine or isomer	C_21_H_24_N_2_O_3_	17.45	353.18652		335.1740	291.1498	166.0862	158.0964	144.0808	
10 ^2^	Echitamine	C_22_H_29_N_2_O_4_	18.04	385.21273		367.2022	349.1909	310.1435	250.1225	220.1121	[30]
11	Sweroside or isomer	C_16_H_22_O_9_	18.07	359.13421		197.0810	179.0705	151.0753	127.0392	111.0807	
12	Unidentified N-formylalkaloid 1 isomer 2	C_22_H_24_N_2_O_5_	18.25	397.17635		369.1808	337.1544	299.1390	256.0969	224.0708	
13	Nα-Formyl-12-methoxyechitamidine	C_22_H_26_N_2_O_5_	18.31	399.19200		371.1963	339.1702	299.1392	267.1128	198.0916	[31]
14	5-O-Feruloylquinic acid	C_17_H_20_O_9_	18.47		367.10291	193.0502	191.0555	173.0444	134.0362	93.0330	
15 ^1^	4-Coumaric acid	C_9_H_8_O_3_	18.50		163.03952	119.0488	93.0331				
16	Vallesamine or isomer	C_20_H_24_N_2_O_3_	18.73	341.18652		309.1596	237.1022	209.1075	194.0965	168.0806	
17	Loliolide	C_11_H_16_O_3_	20.10	197.11777		179.1068	161.0961	135.1170	133.1014	107.0859	
18	Unidentified N-formylalkaloid 2	C_22_H_24_N_2_O_4_	20.41	381.18143		353.1858	339.1705	321.1596	263.1185	212.0939	
19	Secologanoside methyl ester	C_17_H_24_O_11_	20.72		403.12404	371.0987	223.0608	179.0550	165.0548	121.0281	
20	Lagumicine or isomer	C_20_H_22_N_2_O_4_	21.02	355.16578		337.1545	214.0863	200.0708	182.0602	154.0652	
21	Akuammicine	C_20_H_22_N_2_O_2_	21.58	323.17596		294.1490	291.1494	280.1331	263.1543	234.1281	
22	Unidentified alkaloid isomer 1	C_21_H_24_N_2_O_4_	21.67	369.18143		337.1547	309.1600	299.1390	267.1127	224.0703	
23	Echitamidine	C_20_H_24_N_2_O_3_	21.98	341.18652		323.1758	309.1598	202.0864	142.0653	140.1071	[30]
24	Unidentified alkaloid isomer 2	C_21_H_24_N_2_O_4_	22.17	369.18143		337.1547	309.1602	299.1391	256.0969	224.0710	
25	Akuammicine isomer 1	C_20_H_22_N_2_O_2_	22.83	323.17596		291.1493	280.1337	263.1538	249.1389	234.1289	
26	Tubotaiwine	C_20_H_24_N_2_O_2_	23.02	325.19160		293.1648	265.1340	236.1435	222.1282	194.0967	
27	Akuammicine isomer 2	C_20_H_22_N_2_O_2_	23.25	323.17596		291.1493	280.1341	263.1549	249.1388	234.1277	
28	17-O-Acetyl-N-demethylechitamine or isomer	C_23_H_28_N_2_O_5_	23.29	413.20765		395.1972	353.1858	335.1755	292.1332	232.1121	
29 ^1^	Isoquercitrin (Quercetin-3-O-glucoside)	C_21_H_20_O_12_	23.44		463.08765	301.0358	300.0280	271.0252	255.0288	151.0025	
30 ^1^	Rutin (Quercetin-3-O-rutinoside)	C_27_H_30_O_16_	23.52		609.14557	301.0355	300.0280	271.0251	255.0302	151.0031	
31	Voacangine	C_22_H_28_N_2_O_3_	24.93	369.21782		337.1909	309.1598	266.1167	252.1030		[33]
32 ^1^	Quercitrin (Quercetin-3-O-rhamnoside)	C_21_H_20_O_11_	25.01		447.09274	301.0359	300.0279	271.0252	255.0289	151.0030	
33	Akuammidine	C_21_H_24_N_2_O_3_	25.02	353.18652		321.1599	310.1441	293.1654	264.1377	250.1245	[33]
34	Dihydroakuammidine or isomer	C_21_H_26_N_2_O_3_	25.92	355.20217		323.1753	295.1441	266.1550	252.1387	224.1069	
35	Dihydroactinidiolide	C_11_H_16_O_2_	27.15	181.12286		163.1117	145.1014	135.1170	121.1013	107.0859	
36	9-Hydroxyoctadecatrienoic acid	C_18_H_30_O_3_	40.14		293.21167	275.2021	231.2113	171.1016	121.1009	59.0130	
37	13-Hydroxyoctadecatrienoic acid	C_18_H_30_O_3_	40.25		293.21167	275.2021	235.1700	223.1336	195.1384	179.1437	
38	Linoleamide	C_18_H_33_NO	44.45	280.26404		263.2369	245.2264	109.1015	95.0860	81.0704	
39	Oleamide	C_18_H_35_NO	45.70	282.27969		265.2530	247.2418	135.1171	83.0861	69.0705	
40	Pheophytin A	C_55_H_74_N_4_O_5_	65.78	871.57375		593.2762	533.2550	460.2255			

^1^ Confirmed by standard. ^2^ [M]^+^.

**Table 4:** Chemical characterization of leaves—MAC-MeOH (not stirred).

No.	Name	Formula	Rt	[M + H]^+^	[M − H]^−^	Fragment 1	Fragment 2	Fragment 3	Fragment 4	Fragment 5	Literature
1	Quinic acid	C_7_H_12_O_6_	1.26		191.05557	173.0446	171.0292	127.0387	111.0438	93.0330	
2	Unidentified dihydroxybenzoic acid derivative	C_22_H_20_O_12_	13.64		475.08765	299.0774	153.0181	137.0232	109.0281		
3 ^1^	Chlorogenic acid (3-O-Caffeoylquinic acid)	C_16_H_18_O_9_	14.88	355.10291		163.0390	145.0285	135.0442	117.0337	89.0390	
4	Caffeic acid	C_9_H_8_O_4_	15.19		179.03444	135.0440	107.0489				
5	Loganic acid	C_16_H_24_O_10_	15.76		375.12913	213.0764	169.0860	151.0753	113.0230	69.0330	
6	Vallesamine or isomer	C_20_H_24_N_2_O_3_	15.77	341.18652		309.1596	236.1069	208.1127	194.0962	168.0807	
7	Swertiamarin or isomer	C_16_H_22_O_10_	16.73	375.12912		213.0757	195.0653	177.0546	151.0390	107.0495	
8	Unidentified N-formylalkaloid 1 isomer 1	C_22_H_24_N_2_O_5_	17.27	397.17635		369.1810	337.1546	299.1392	267.1127	224.0704	
9	Lochnericine or isomer	C_21_H_24_N_2_O_3_	17.29	353.18652		335.1751	291.1490	166.0862	158.0964	144.0807	
10	Secologanoside	C_16_H_22_O_11_	17.33		389.10839	345.1191	209.0452	183.0656	165.0546	69.0330	
11	5-O-(4-Coumaroyl)quinic acid	C_16_H_18_O_8_	17.47		337.09235	191.0555	173.0445	163.0390	119.0489	93.0331	
12	Picralinal or isomer	C_21_H_22_N_2_O_4_	17.59	367.16578		349.1923	339.1703	307.1440	269.1285	180.1020	
13	Quercetin-O-hexoside-O-rutinoside isomer 1	C_33_H_40_O_21_	17.70		771.19839	609.1468	462.0807	301.0357	300.0279	299.0201	
14 ^2^	Echitamine	C_22_H_29_N_2_O_4_	17.89	385.21273		367.2024	349.1920	310.1432	250.1231	220.1122	[30]
15	Picralinal or isomer	C_21_H_22_N_2_O_4_	17.94	367.16578		349.1930	339.1703	307.1441	269.1285	194.0601	
16	Quebrachidine or isomer	C_21_H_24_N_2_O_3_	17.96	353.18652		335.1750	324.1594	303.1491	293.1651	275.1537	
17	Quercetin-O-hexoside-O-rutinoside isomer 2	C_33_H_40_O_21_	17.99		771.19839	609.1483	463.0887	462.0813	301.0358	299.0201	
18	Sweroside or isomer	C_16_H_22_O_9_	18.02	359.13421		197.0810	179.0703	151.0754	127.0392	111.0808	
19	Unidentified N-formylalkaloid 1 isomer 2	C_22_H_24_N_2_O_5_	18.06	397.17635		369.1809	337.1545	299.1390	256.0968	224.0708	
20	4-O-(4-Coumaroyl)quinic acid	C_16_H_18_O_8_	18.10		337.09235	191.0555	173.0445	163.0389	119.0488	93.0330	
21	Nα-Formyl-12-methoxyechitamidine	C_22_H_26_N_2_O_5_	18.12	399.19200		371.1962	339.1701	299.1392	267.1130	198.0914	[31]
22 ^1^	4-Coumaric acid	C_9_H_8_O_3_	18.50		163.03952	119.0488	93.0332				
23	Vallesamine or isomer	C_20_H_24_N_2_O_3_	18.52	341.18652		309.1597	237.1018	209.1073	194.0965	168.0808	
24	5-O-Feruloylquinic acid	C_17_H_20_O_9_	18.54		367.10291	193.0499	191.0555	173.0445	134.0362	93.0331	
25	N-Formylechitamidine	C_21_H_24_N_2_O_4_	18.85	369.18143		351.1705	341.1858	309.1598	202.0864		[32]
26	5-O-(4-Coumaroyl)quinic acid cis isomer	C_16_H_18_O_8_	19.69		337.09235	191.0553	173.0446	163.0387	119.0488	93.0330	
27	Loliolide	C_11_H_16_O_3_	20.03	197.11777		179.1067	161.0961	135.1169	133.1014	107.0858	
28	Unidentified N-formylalkaloid 2	C_22_H_24_N_2_O_4_	20.20	381.18143		353.1858	339.1711	321.1595	263.1182	212.0934	
29	Secologanol	C_17_H_26_O_10_	20.35	391.16043		229.1070	211.0965	193.0862	179.0704	167.0704	
30	Quercecin-O-hexosylhexoside	C_27_H_30_O_17_	20.66		625.14048	301.0359	300.0278	271.0250	255.0298	151.0024	
31	Secologanoside methyl ester	C_17_H_24_O_11_	20.73		403.12404	371.0990	223.0611	179.0550	165.0546	121.0281	
32	Lagumicine or isomer	C_20_H_22_N_2_O_4_	20.90	355.16578		337.1546	214.0862	200.0708	182.0602	154.0651	
33	Akuammicine	C_20_H_22_N_2_O_2_	21.38	323.17596		294.1488	291.1493	280.1334	263.1543	234.1279	
34	Unidentified alkaloid isomer 1	C_21_H_24_N_2_O_4_	21.47	369.18143		337.1545	309.1595	299.1392	267.1129	224.0714	
35	Cinchonain I isomer 1	C_24_H_20_O_9_	21.70		451.10291	341.0668	231.0295	217.0138	189.0186	177.0184	
36	Echitamidine	C_20_H_24_N_2_O_3_	21.73	341.18652		323.1762	309.1596	202.0863	142.0652	140.1070	[30]
37	N-Methylakuammicine	C_21_H_24_N_2_O_2_	21.96	337.19161		309.1580	305.1648	294.1504	277.1697	263.1541	
38	Unidentified alkaloid isomer 2	C_21_H_24_N_2_O_4_	22.01	369.18143		337.1545	309.1590	299.1391	256.0968	224.0708	
39	Akuammicine isomer N-methyl derivative	C_21_H_24_N_2_O_2_	22.49	337.19161		305.1648	277.1702	263.1542	248.1074	222.1277	
40	Cinchonain I isomer 2	C_24_H_20_O_9_	22.69		451.10291	341.0668	231.0294	217.0138	189.0187	177.0183	
41	Akuammicine isomer 1	C_20_H_22_N_2_O_2_	22.71	323.17596		291.1491	280.1332	263.1538	249.1385	234.1290	
42	Tubotaiwine	C_20_H_24_N_2_O_2_	22.87	325.19160		293.1647	265.1341	236.1434	222.1282	194.0964	
43	Akuammicine isomer 2	C_20_H_22_N_2_O_2_	23.09	323.17596		291.1491	280.1328	263.1541	249.1385	234.1309	
44	17-O-Acetyl-N-demethylechitamine or isomer	C_23_H_28_N_2_O_5_	23.15	413.20765		395.1971	353.1856	335.1757	292.1333	232.1120	
45 ^1^	Isoquercitrin (Quercetin-3-O-glucoside)	C_21_H_20_O_12_	23.45		463.08765	301.0357	300.0278	271.0249	255.0299	151.0024	
46 ^1^	Rutin (Quercetin-3-O-rutinoside)	C_27_H_30_O_16_	23.54		609.14557	301.0357	300.0278	271.0250	255.0299	151.0024	
47	Di-O-caffeoylquinic acid	C_25_H_24_O_12_	24.65		515.11896	353.0880	335.0790	191.0553	179.0340	173.0444	
48	Voacangine	C_22_H_28_N_2_O_3_	24.75	369.21782		337.1907	309.1591	266.1159	252.1027		[33]
49	Akuammidine	C_21_H_24_N_2_O_3_	24.91	353.18652		321.1598	310.1444	293.1647	264.1374	250.1248	[33]
50 ^1^	Quercitrin (Quercetin-3-O-rhamnoside)	C_21_H_20_O_11_	25.03		447.09274	301.0356	300.0278	271.0250	255.0299	151.0024	
51	Dihydroakuammidine or isomer	C_21_H_26_N_2_O_3_	25.79	355.20217		323.1753	295.1441	266.1559	252.1392	224.1068	
52	Dimethoxy-tetrahydroxy(iso)flavone	C_17_H_14_O_8_	26.52		345.06105	330.0384	315.0151	287.0199	271.0262	243.0297	
53 ^1^	Quercetin (3,3′,4′,5,7-Pentahydroxyflavone)	C_15_H_10_O_7_	27.56		301.03484	273.0393	178.9981	151.0024	121.0281	107.0125	
54	Dimethoxy-trihydroxy(iso)flavone	C_17_H_14_O_7_	33.32		329.06613	314.0436	299.0200	285.0400	271.0251	243.0299	
55	Dihydroxy-trimethoxy(iso)flavone isomer 1	C_18_H_16_O_7_	35.18		343.08178	328.0594	313.0359	285.0395	269.0471	257.0465	
56	Dihydroxy-trimethoxy(iso)flavone isomer 2	C_18_H_16_O_7_	35.52		343.08178	328.0589	313.0358	285.0405	269.0451	257.0447	
57	9-Hydroxyoctadecatrienoic acid	C_18_H_30_O_3_	40.17		293.21167	275.2022	231.2119	171.1016	121.1009	59.0125	
58	13-Hydroxyoctadecatrienoic acid	C_18_H_30_O_3_	40.27		293.21167	275.2021	235.1699	223.1334	195.1384	179.1430	
59	Linoleamide	C_18_H_33_NO	44.44	280.26404		263.2371	245.2262	109.1015	95.0860	81.0705	
60	Oleamide	C_18_H_35_NO	45.68	282.27969		265.2525	247.2418	135.1168	83.0860	69.0705	
61	Pheophytin A	C_55_H_74_N_4_O_5_	65.73	871.57375		593.2762	533.2551	460.2258			

^1^ Confirmed by standard. ^2^ [M]^+^.

**Table 5:** Chemical characterization of Stem bark—infusion.

No.	Name	Formula	Rt	[M + H]^+^	[M − H]^−^	Fragment 1	Fragment 2	Fragment 3	Fragment 4	Fragment 5	Literature
1	Quinic acid	C_7_H_12_O_6_	1.25		191.05557	173.0444	171.0289	127.0387	111.0437	93.0330	
2	Neochlorogenic acid (5-O-Caffeoylquinic acid)	C_16_H_18_O_9_	10.29	355.10291		163.0389	145.0284	135.0442	117.0337	89.0390	
3	3-O-(4-Coumaroyl)quinic acid	C_16_H_18_O_8_	13.33		337.09235	191.0553	173.0444	163.0389	119.0488	93.0329	
4 ^1^	Chlorogenic acid (3-O-Caffeoylquinic acid)	C_16_H_18_O_9_	14.90	355.10291		163.0390	145.0284	135.0442	117.0338	89.0389	
5	3-O-Feruloylquinic acid	C_17_H_20_O_9_	15.14		367.10291	193.0499	191.0556	173.0445	134.0361	93.0331	
6	Loganic acid	C_16_H_24_O_10_	15.72		375.12913	213.0763	169.0860	151.0754	113.0230	69.0330	
7	Vallesamine or isomer	C_20_H_24_N_2_O_3_	15.75	341.18652		309.1598	236.1071	208.1126	194.0968	168.0807	
8	Chryptochlorogenic acid (4-O-Caffeoylquinic acid)	C_16_H_18_O_9_	16.15	355.10291		163.0389	145.0285	135.0442	117.0337	89.0390	
9	Swertiamarin or isomer	C_16_H_22_O_10_	16.75	375.12912		213.0757	195.0654	177.0546	151.0390	107.0495	
10	Unidentified N-formylalkaloid 1 isomer 1	C_22_H_24_N_2_O_5_	17.06	397.17635		369.1811	337.1546	299.1391	267.1130	224.0709	
11	Lochnericine or isomer	C_21_H_24_N_2_O_3_	17.16	353.18652		335.1754	291.1495	166.0863	158.0965	144.0808	
12	Secologanoside	C_16_H_22_O_11_	17.32		389.10839	345.1191	209.0453	183.0654	165.0546	69.0330	
13 ^2^	Echitamine	C_22_H_29_N_2_O_4_	17.33	385.21273		367.2018	349.1896	310.1437	250.1225	220.1120	[30]
14	5-O-(4-Coumaroyl)quinic acid	C_16_H_18_O_8_	17.48		337.09235	191.0553	173.0444	163.0388	119.0488	93.0330	
15	Picralinal or isomer	C_21_H_22_N_2_O_4_	17.94	367.16578		349.1930	339.1703	307.1441	269.1285	194.0601	
16	Sweroside or isomer	C_16_H_22_O_9_	18.02	359.13421		197.0811	179.0704	151.0754	127.0392	111.0808	
17	4-O-(4-Coumaroyl)quinic acid	C_16_H_18_O_8_	18.10		337.09235	191.0551	173.0444	163.0390	119.0488	93.0330	
18	Unidentified N-formylalkaloid 1 isomer 2	C_22_H_24_N_2_O_5_	18.12	397.17635		369.1810	337.1546	299.1389	256.0966	224.0708	
19	Nα-Formyl-12-methoxyechitamidine	C_22_H_26_N_2_O_5_	18.17	399.19200		371.1965	339.1703	299.1392	267.1126	198.0917	[31]
20 ^1^	4-Coumaric acid	C_9_H_8_O_3_	18.46		163.03952	119.0488	93.0333				
21	Vallesamine or isomer	C_20_H_24_N_2_O_3_	18.48	341.18652		309.1596	237.1027	209.1079	194.0961	168.0806	
22	5-O-Feruloylquinic acid	C_17_H_20_O_9_	18.50		367.10291	193.0500	191.0554	173.0445	134.0362	93.0330	
23	N-Formylechitamidine	C_21_H_24_N_2_O_4_	18.91	369.18143		351.1702	341.1859	309.1596	202.0861		[32]
24	4-O-Feruloylquinic acid	C_17_H_20_O_9_	19.00		367.10291	193.0500	191.0555	173.0445	134.0361	93.0330	
25	Loganin	C_17_H_26_O_10_	19.03	391.16043		229.1071	211.0966	197.0812	179.0704	109.0651	
26	Loliolide	C_11_H_16_O_3_	20.06	197.11777		179.1068	161.0960	135.1170	133.1014	107.0859	
27	Venalstonine or isomer	C_21_H_24_N_2_O_2_	20.26	337.19161		305.1649	294.1490	277.1698	234.1279	196.0996	
28	Unidentified N-formylalkaloid 2	C_22_H_24_N_2_O_4_	20.36	381.18143		353.1859	339.1691	321.1598	263.1185	212.0945	
29	Secologanoside methyl ester	C_17_H_24_O_11_	20.72		403.12404	371.0988	223.0607	179.0547	165.0545	121.0281	
30	Akuammicine	C_20_H_22_N_2_O_2_	21.42	323.17596		294.1490	291.1493	280.1322	263.1541	234.1304	
31	Unidentified alkaloid isomer 1	C_21_H_24_N_2_O_4_	21.66	369.18143		337.1545	309.1601	299.1392	267.1126	224.0701	
32	Echitamidine	C_20_H_24_N_2_O_3_	21.93	341.18652		323.1738	309.1594	202.0864	142.0653	140.1071	[30]
33	Unidentified alkaloid isomer 2	C_21_H_24_N_2_O_4_	22.13	369.18143		337.1546	309.1597	299.1391	256.0969	224.0708	
34	Unidentified alkaloid 2	C_24_H_30_N_2_O_5_	22.27	427.22330		409.2123	352.1542	292.1332	250.1227	232.1121	
35	7-Deoxyloganic acid	C_16_H_24_O_9_	22.36		359.13421	197.0813	153.0909	135.0803	109.0645	89.0229	
36	Akuammicine isomer 1	C_20_H_22_N_2_O_2_	22.74	323.17596		291.1494	280.1335	263.1538	249.1387	234.1281	
37	Tubotaiwine	C_20_H_24_N_2_O_2_	22.94	325.19160		293.1648	265.1342	236.1444	222.1281	194.0965	
38	Akuammicine isomer 2	C_20_H_22_N_2_O_2_	23.21	323.17596		291.1494	280.1336	263.1546	249.1387	234.1281	
39	Boonein	C_9_H_14_O_3_	23.23		169.08647	151.0751	141.0915	125.0957			[34]
40	17-O-Acetyl-N-demethylechitamine or isomer	C_23_H_28_N_2_O_5_	23.24	413.20765		395.1967	353.1859	335.1765	292.1330	232.1123	
41	Di-O-caffeoylquinic acid	C_25_H_24_O_12_	24.62		515.11896	353.0883	335.0790	191.0554	179.0341	173.0445	
42	Dimethoxy-trihydroxy(iso)flavone	C_17_H_14_O_7_	33.34		329.06613	314.0438	299.0200	285.0400	271.0247	243.0299	
43	Emodin	C_15_H_10_O_5_	39.63		269.04500	241.0501	225.0552	197.0601	210.0318	181.0649	
44	9-Hydroxyoctadecatrienoic acid	C_18_H_30_O_3_	40.14		293.21167	275.2021	231.2109	171.1016	121.1009	59.0125	
45	13-Hydroxyoctadecatrienoic acid	C_18_H_30_O_3_	40.30		293.21167	275.2020	235.1701	223.1335	195.1383	179.1429	
46	Linoleamide	C_18_H_33_NO	44.45	280.26404		263.2367	245.2264	109.1014	95.0859	81.0704	
47	Oleamide	C_18_H_35_NO	45.72	282.27969		265.2527	247.2418	135.1169	83.0860	69.0705	

^1^ Confirmed by standard. ^2^ [M]^+^.

**Table 6:** Chemical characterization of stem bark—MAC-EA (not stirred).

No.	Name	Formula	Rt	[M + H]^+^	[M − H]^−^	Fragment 1	Fragment 2	Fragment 3	Fragment 4	Fragment 5	Literature
1	Quinic acid	C_7_H_12_O_6_	1.27		191.05557	173.0447	171.0288	127.0388	111.0437	93.0330	
2 ^1^	Chlorogenic acid (3-O-Caffeoylquinic acid)	C_16_H_18_O_9_	14.91	355.10291		163.0390	145.0285	135.0442	117.0338	89.0389	
3	Loganic acid	C_16_H_24_O_10_	15.77		375.12913	213.0762	169.0859	151.0754	113.0229	69.0330	
4	Swertiamarin or isomer	C_16_H_22_O_10_	16.73	375.12912		213.0757	195.0654	177.0548	151.0390	107.0495	
5	Secologanoside	C_16_H_22_O_11_	17.34		389.10839	345.1187	209.0451	183.0653	165.0545	69.0330	
6	5-O-(4-Coumaroyl)quinic acid	C_16_H_18_O_8_	17.46		337.09235	191.0553	173.0439	163.0383	119.0489	93.0328	
7 ^2^	Echitamine	C_22_H_29_N_2_O_4_	17.83	385.21273		367.2011	349.1912	310.1437	250.1226	220.1121	[30]
8	Sweroside or isomer	C_16_H_22_O_9_	18.05	359.13421		197.0810	179.0704	151.0755	127.0392	111.0808	
9	Nα-Formyl-12-methoxyechitamidine	C_22_H_26_N_2_O_5_	18.31	399.19200		371.1964	339.1703	299.1392	267.1131	198.0914	[31]
10	5-O-Feruloylquinic acid	C_17_H_20_O_9_	18.50		367.10291	193.0498	191.0554	173.0445	134.0362	93.0330	
11 ^1^	4-Coumaric acid	C_9_H_8_O_3_	18.52		163.03952	119.0487	93.0328				
12	Vallesamine or isomer	C_20_H_24_N_2_O_3_	18.58	341.18652		309.1598	237.1021	209.1074	194.0965	168.0806	
13	Loganin	C_17_H_26_O_10_	18.97	391.16043		229.1070	211.0965	197.0812	179.0704	109.0652	
14	Loliolide	C_11_H_16_O_3_	20.07	197.11777		179.1068	161.0961	135.1170	133.1013	107.0859	
15	Venalstonine or isomer	C_21_H_24_N_2_O_2_	20.38	337.19161		305.1649	294.1485	277.1699	234.1279	196.0998	
16	Secologanoside methyl ester	C_17_H_24_O_11_	20.73		403.12404	371.0984	223.0609	179.0549	165.0545	121.0281	
17	Echitamidine	C_20_H_24_N_2_O_3_	22.03	341.18652		323.1751	309.1603	202.0864	142.0653	140.1071	[30]
18	7-Deoxyloganic acid	C_16_H_24_O_9_	22.35		359.13421	197.0813	153.0909	135.0802	109.0644	89.0228	
19	Unidentified alkaloid 2	C_24_H_30_N_2_O_5_	22.37	427.22330		409.2125	352.1544	292.1333	250.1228	232.1122	
20	Tubotaiwine	C_20_H_24_N_2_O_2_	22.95	325.19160		293.1648	265.1346	236.1428	222.1279	194.0959	
21	17-O-Acetyl-N-demethylechitamine or isomer	C_23_H_28_N_2_O_5_	23.22	413.20765		395.1960	353.1850	335.1763	292.1329	232.1122	
22	Di-O-caffeoylquinic acid	C_25_H_24_O_12_	24.63		515.11896	353.0882	335.0790	191.0553	179.0339	173.0444	
23	Dimethoxy-trihydroxy(iso)flavone	C_17_H_14_O_7_	33.35		329.06613	314.0434	299.0199	285.0410	271.0249	243.0290	
24	Linoleamide	C_18_H_33_NO	44.47	280.26404		263.2370	245.2264	109.1015	95.0860	81.0705	
25	Oleamide	C_18_H_35_NO	45.71	282.27969		265.2525	247.2424	135.1170	83.0860	69.0705	
26	Pheophytin A	C_55_H_74_N_4_O_5_	65.51	871.57375		593.2759	533.2547	460.2255			

^1^ Confirmed by standard. ^2^ [M]^+^.

**Table 7:** Chemical characterization of stem bark—MAC-MEOH (not stirred).

No.	Name	Formula	Rt	[M + H]^+^	[M − H]^−^	Fragment 1	Fragment 2	Fragment 3	Fragment 4	Fragment 5	Literature
1	Quinic acid	C_7_H_12_O_6_	1.25		191.05557	173.0443	171.0289	127.0388	111.0435	93.0330	
2 ^1^	Chlorogenic acid (3-O-Caffeoylquinic acid)	C_16_H_18_O_9_	14.88	355.10291		163.0389	145.0284	135.0442	117.0337	89.0390	
3	Loganic acid	C_16_H_24_O_10_	15.69		375.12913	213.0762	169.0859	151.0750	113.0229	69.0329	
4	Swertiamarin or isomer	C_16_H_22_O_10_	16.69	375.12912		213.0757	195.0653	177.0546	151.0389	107.0495	
5	Unidentified N-formylalkaloid 1 isomer 1	C_22_H_24_N_2_O_5_	16.86	397.17635		369.1806	337.1543	299.1389	267.1126	224.0704	
6^2^	Echitamine	C_22_H_29_N_2_O_4_	16.92	385.21273		367.2010	349.1913	310.1436	250.1224	220.1118	[30]
7	Secologanoside	C_16_H_22_O_11_	17.33		389.10839	345.1187	209.0447	183.0656	165.0544	69.0329	
8	5-O-(4-Coumaroyl)quinic acid	C_16_H_18_O_8_	17.48		337.09235	191.0552	173.0444	163.0388	119.0487	93.0330	
9	Sweroside or isomer	C_16_H_22_O_9_	17.86	359.13421		197.0808	179.0702	151.0753	127.0391	111.0807	
10	Unidentified N-formylalkaloid 1 isomer 2	C_22_H_24_N_2_O_5_	18.05	397.17635		369.1807	337.1545	299.1389	256.0966	224.0707	
11	Nα-Formyl-12-methoxyechitamidine	C_22_H_26_N_2_O_5_	18.11	399.19200		371.1964	339.1702	299.1392	267.1128	198.0919	[31]
12	Vallesamine or isomer	C_20_H_24_N_2_O_3_	18.42	341.18652		309.1596	237.1024	209.1073	194.0963	168.0807	
13 ^1^	4-Coumaric acid	C_9_H_8_O_3_	18.49		163.03952	119.0487	93.0331				
14	5-O-Feruloylquinic acid	C_17_H_20_O_9_	18.50		367.10291	193.0498	191.0554	173.0444	134.0360	93.0330	
15	N-Formylechitamidine	C_21_H_24_N_2_O_4_	18.82	369.18143		351.1700	341.1859	309.1596	202.0860		[32]
16	Loganin	C_17_H_26_O_10_	18.89	391.16043		229.1071	211.0969	197.0800	179.0705	109.0650	
17	Loliolide	C_11_H_16_O_3_	20.04	197.11777		179.1068	161.0960	135.1170	133.1014	107.0859	
18	Venalstonine or isomer	C_21_H_24_N_2_O_2_	20.07	337.19161		305.1647	294.1482	277.1697	234.1274	196.0992	
19	Unidentified N-formylalkaloid 2	C_22_H_24_N_2_O_4_	20.28	381.18143		353.1859	339.1705	321.1596	263.1181	212.0936	
20	Secologanoside methyl ester	C_17_H_24_O_11_	20.71		403.12404	371.0983	223.0604	179.0547	165.0545	121.0280	
21	Akuammicine	C_20_H_22_N_2_O_2_	21.46	323.17596		294.1487	291.1491	280.1331	263.1540	234.1284	
22	Echitamidine	C_20_H_24_N_2_O_3_	21.75	341.18652		323.1754	309.1594	202.0863	142.0652	140.1071	[30]
23	Unidentified alkaloid 2	C_24_H_30_N_2_O_5_	22.06	427.22330		409.2113	352.1541	292.1334	250.1222	232.1119	
24	7-Deoxyloganic acid	C_16_H_24_O_9_	22.35		359.13421	197.0812	153.0908	135.0802	109.0644	89.0228	
25	Akuammicine isomer 1	C_20_H_22_N_2_O_2_	22.62	323.17596		291.1491	280.1329	263.1538	249.1384	234.1269	
26	Tubotaiwine	C_20_H_24_N_2_O_2_	22.81	325.19160		293.1646	265.1328	236.1436	222.1281	194.0963	
27	Akuammicine isomer 2	C_20_H_22_N_2_O_2_	23.06	323.17596		291.1491	280.1329	263.1538	249.1384	234.1269	
28	Boonein	C_9_H_14_O_3_	23.12		169.08647	151.0752	141.0909	125.0959			[34]
29	17-O-Acetyl-N-demethylechitamine or isomer	C_23_H_28_N_2_O_5_	23.14	413.20765		395.1974	353.1853	335.1755	292.1328	232.1122	
30	Di-O-caffeoylquinic acid	C_25_H_24_O_12_	24.62		515.11896	353.0878	335.0742	191.0553	179.0339	173.0443	
31	Dimethoxy-trihydroxy(iso)flavone	C_17_H_14_O_7_	33.34		329.06613	314.0434	299.0197	285.0404	271.0247	243.0296	
32	9-Hydroxyoctadecatrienoic acid	C_18_H_30_O_3_	40.16		293.21167	275.2019	231.2110	171.1015	121.1008	59.0125	
33	13-Hydroxyoctadecatrienoic acid	C_18_H_30_O_3_	40.26		293.21167	275.2017	235.1699	223.1332	195.1384	179.1441	
34	Linoleamide	C_18_H_33_NO	44.44	280.26404		263.2368	245.2262	109.1015	95.0860	81.0704	
35	Oleamide	C_18_H_35_NO	45.70	282.27969		265.2524	247.2424	135.1169	83.0860	69.0705	
36	Pheophytin A	C_55_H_74_N_4_O_5_	65.49	871.57375		593.2759	533.2549	460.2255			

^1^ Confirmed by standard. ^2^ [M]^+^.

**Table 8:** Antioxidant properties of the studied extracts *.

Parts	Extraction Methods/Solvent	**DPPH^•^** **(mg TE/g)**	**ABTS^•+^** **(mg TE/g)**	CUPRAC (mg TE/g)	FRAP (mg TE/g)	Metal Chelating (mg EDTAE/g)
Leaves	Infusion-water	74.70 ± 1.27 ^a^	131.50 ± 2.34 ^a^	140.57 ± 0.37 ^d^	109.35 ± 1.48 ^a^	13.42 ± 2.06 ^e^
	MAC-EA	13.96 ± 0.87 ^f^	36.78 ± 1.73 ^f^	82.79 ± 1.68 ^f^	41.54 ± 0.43 ^f^	31.51 ± 1.05 ^a^
	MAC-MeOH	55.14 ± 0.44 ^c^	109.24 ± 3.07 ^c^	146.71 ± 3.80 ^c^	87.37 ± 1.42 ^d^	26.07 ± 0.52 ^b^
	MAC-EA (not stirred)	18.40 ± 0.64 ^e^	43.67 ± 2.11 ^e^	87.72 ± 1.10 ^f^	38.03 ± 0.86 ^g^	24.32 ± 1.11 ^bc^
	MAC-MeOH (not stirred)	61.23 ± 1.46 ^b^	123.70 ± 0.27 ^b^	163.91 ± 2.20 ^a^	100.86 ± 0.40 ^b^	22.67 ± 0.50 ^cd^
	SE-EA	27.99 ± 1.11 ^d^	88.67 ± 1.26 ^d^	118.54 ± 0.86 ^e^	61.48 ± 1.78 ^e^	21.17 ± 0.63 ^d^
	SE-MeOH	61.32 ± 0.47 ^b^	127.63 ± 1.65 ^ab^	155.36 ± 1.10 ^b^	97.05 ± 0.74 ^c^	21.90 ± 0.41 ^cd^
Stem bark	Infusion-water	33.82 ± 0.66 ^b^	59.94 ± 1.70 ^c^	74.80 ± 0.56 ^c^	55.48 ± 1.17 ^ab^	36.71 ± 0.14 ^a^
	MAC-EA	4.54 ± 0.94 ^c^	na	76.51 ± 1.11 ^c^	40.41 ± 0.80 ^c^	na
	MAC-MeOH	35.41 ± 0.47 ^b^	69.24 ± 2.82 ^b^	94.89 ± 1.59 ^b^	53.94 ± 2.18 ^b^	22.21 ± 1.93 ^b^
	MAC-EA (not stirred)	5.88 ± 0.76 ^c^	na	74.37 ± 2.06 ^c^	36.04 ± 0.12 ^d^	na
	MAC- MeOH (not stirred)	37.57 ± 0.74 ^a^	73.69 ± 1.17 ^a^	105.92 ± 4.86 ^a^	54.48 ± 1.67 ^b^	23.77 ± 0.23 ^b^
	SE-EA	5.94 ± 1.07 ^c^	6.04 ± 0.91 ^d^	78.31 ± 0.72 ^c^	36.64 ± 0.15 ^d^	na
	SE-MeOH	34.74 ± 0.48 ^b^	71.22 ± 0.17 ^ab^	101.53 ± 1.80 ^a^	58.03 ± 0.80 ^a^	18.23 ± 0.16 ^c^

* Values expressed are means ± S.D. of three parallel measurements. TE: Trolox equivalent; EDTAE: EDTA equivalent. MAC: Maceration; SE: Soxhlet extraction; EA: Ethyl acetate; MeOH: Methanol, na: not active. Different letters indicate significant differences in the tested extracts of each parts (*p* < 0.05).

**Table 9:** Enzyme inhibitory properties of the studied extracts *.

Parts	Extraction Methods/Solvent	**AChE** **(mg GALAE/g)**	**BChE** **(mg GALAE/g)**	Amylase (mmol ACAE/g)	Glucosidase (mmol ACAE/g)	Tyrosinase (mg KAE/g)
Leaves	Infusion-water	na	6.35 ± 0.18 ^b^	0.17 ± 0.01 ^d^	na	0.72 ± 0.07 ^c^
	MAC-EA	5.02 ± 0.34 ^ab^	4.78 ± 0.11 ^cd^	1.16 ± 0.08 ^a^	na	129.70 ± 2.85 ^ab^
	MAC-MeOH	4.49 ± 0.11 ^b^	3.22 ± 0.92 ^e^	0.91 ± 0.02 ^c^	5.24 ± 0.09 ^a^	132.83 ± 1.04 ^ab^
	MAC-EA (not stirred)	4.45 ± 0.67 ^b^	8.25 ± 0.43 ^a^	1.13 ± 0.06 ^a^	na	127.11 ± 9.18 ^b^
	MAC-MeOH (not stirred)	5.57 ± 0.49 ^a^	6.18 ± 0.19 ^bc^	0.97 ± 0.03 ^bc^	5.27 ± 0.09 ^a^	135.89 ± 0.63 ^ab^
	SE-EA	4.89 ± 0.06 ^ab^	5.19 ± 0.58 ^bc^	1.10 ± 0.09 ^ab^	na	130.08 ± 5.20 ^ab^
	SE-MeOH	5.04 ± 0.34 ^ab^	3.49 ± 0.60 ^de^	0.88 ± 0.02 ^c^	5.04 ± 0.60 ^a^	139.20 ± 0.48 ^a^
Stem bark	Infusion-water	na	10.08 ± 0.29 ^a^	0.20 ± 0.01 ^c^	na	0.43 ± 0.01 ^d^
	MAC-EA	5.02 ± 0.43 ^a^	1.44 ± 0.36 ^d^	1.01 ± 0.08 ^a^	4.71 ± 0.01 ^c^	121.85 ± 1.56 ^b^
	MAC-MeOH	5.34 ± 0.37 ^a^	2.51 ± 0.28 ^d^	0.71 ± 0.06 ^b^	5.40 ± 0.01 ^a^	133.51 ± 1.10 ^a^
	MAC-EA (not stirred)	4.91 ± 0.62 ^a^	1.59 ± 0.03 ^d^	0.98 ± 0.06 ^a^	4.67 ± 0.02 ^d^	123.69 ± 0.53 ^b^
	MAC- MeOH (not stirred)	5.62 ± 0.23 ^a^	4.13 ± 0.65 ^c^	0.80 ± 0.02 ^b^	5.38 ± 0.01 ^a^	135.02 ± 1.20 ^a^
	SE-EA	5.78 ± 0.18 ^a^	6.39 ± 0.47 ^b^	1.04 ± 0.01 ^a^	4.83 ± 0.01 ^b^	94.63 ± 2.53 ^c^
	SE-MeOH	5.67 ± 0.20 ^a^	4.38 ± 0.66 ^c^	0.78 ± 0.03 ^b^	5.41 ± 0.01 ^a^	134.63 ± 0.56 ^a^

* Values expressed are means ± S.D. of three parallel measurements. GALAE: Galatamine equivalent; KAE: Kojic acid equivalent; ACAE: Acarbose equivalent; MAC: Maceration; SE: Soxhlet extraction; EA: Ethyl acetate; MeOH: Methanol, na: not active. Different letters indicate significant differences in the tested extracts of each part (*p* < 0.05).

**Table 10:** Effects induced by *Alstonia boonei* extracts on cell viability and LPS-induced cytokine release (IL-6, TNF-α and IL-1β) in macrophages. Macrophages were exposed for 24 h to 25, 50 and 100 μg/mL of extracts. ^a^ is for *p* < 0.05, ^b^ is for *p* < 0.01, ^c^ is for *p* < 0.001 and ^d^ is for *p* < 0.0001 vs. LPS. * is for absolute values in pg/mL. Data were statistically analyzed using One-Way ANOVA followed by Dunnett’s multiple comparisons test (n = 6 replicates of 2 separate sets of experiments). Results are expressed as mean ± SEM.

Control and Extracts	Sample (μg/mL)	Cell Viability-MTT (%) (Mean ± SEM)	IL-6 Release (%) (Mean ± SEM)	TNF-α Release (%) (Mean ± SEM)	IL-1β Release (%) (Mean ± SEM)
LPS	0.1		583.3 ± 61.3 *	916.7 ± 48.7 *	1033.3 ± 68.0 *
Dexamethasone	0.04		150.0 ± 26.4 *	189.2 ± 36.8 *	330.0 ± 88.5 *
Leaves—infusion	25	97.0 ± 1.2	85.4 ± 4.4	92.0 ± 4.7	77.8 ± 5.9
	50	94.3 ± 3.1	73.5 ± 7.0 ^a^	69.7 ± 3.2 ^a^	61.8 ± 4.4 ^a^
	100	89.9 ± 4.7	64.0 ± 4.7 ^b^	52.4 ± 6.9 ^c^	53.2 ± 5.9 ^b^
Leaves—MAC-EA (not stirred)	25	99.1 ± 2.1	91.7 ± 3.7	92.0 ± 3.0	93.3 ± 3.0
	50	83.3 ± 5.9 ^b^	87.2 ± 3.9	85.1 ± 5.1	88.9 ± 3.8
	100	73.7 ± 2.7 ^c^	73.1 ± 3.6 ^a^	66.3 ± 5.2 ^b^	69.5 ± 4.8 ^b^
Leaves—MAC-MeOH (not stirred)	25	94.5 ± 2.6	90.6 ± 2.0	82.3 ± 4.2	82.2 ± 5.0
	50	96.5 ± 2.4	68.4 ± 2.2 ^c^	70.0 ± 4.6 ^b^	64.3 ± 4.5 ^b^
	100	85.9 ± 3.7 ^b^	62.9 ± 1.4 ^c^	62.9 ± 4.1 ^c^	44.5 ± 5.9 ^d^
Stem bark—Infusion	25	98.9 ± 1.0	68.1 ± 6.7 ^b^	76.6 ± 6.3 ^a^	70.7 ± 7.1 ^a^
	50	96.7 ± 2.1	44.6 ± 5.4 ^d^	50.7 ± 4.6 ^d^	44.2 ± 5.3 ^d^
	100	94.3 ± 3.5	19.0 ± 1.7 ^d^	31.0 ± 2.4 ^d^	23.5 ± 3.0 ^d^
Stem bark—MAC-EA (not stirred)	25	92.9 ± 3.6	94.8 ± 2.0	92.2 ± 1.8	98.3 ± 1.6
	50	83.9 ± 3.2 ^a^	92.4 ± 3.1	86.1 ± 4.3	87.1 ± 3.9
	100	76.1 ± 4.8 ^c^	81.5 ± 5.4 ^a^	71.5 ± 4.2 ^c^	64.3 ± 5.4 ^d^
Stem bark—MAC-MeOH (not stirred)	25	98.6 ± 2.7	66.6 ± 2.6 ^d^	66.2 ± 7.2 ^c^	64.0 ± 6.1 ^d^
	50	92.6 ± 2.2	48.5 ± 4.7 ^d^	44.0 ± 4.0 ^d^	38.4 ± 4.1 ^d^
	100	89.4 ± 3.2 ^a^	29.0 ± 4.1 ^d^	22.9 ± 3.3 ^d^	22.4 ± 2.8 ^d^

## Data Availability

Not applicable.

## Supplementary Materials

The following supporting information can be downloaded at: https://www.mdpi.com/article/10.3390/antiox11112171/s1, Figure S1. Total ion chromatogram of *Alstonia boonei* leaves infusion in positive mode; Figure S2. Total ion chromatogram of *Alstonia boonei* leaves infusion in negative mode; Figure S3. Total ion chromatogram of *Alstonia boonei* leaves maceration-EA in positive mode; Figure S4. Total ion chromatogram of *Alstonia boonei* leaves maceration-EA in negative mode; Figure S5. Total ion chromatogram of *Alstonia boonei* leaves maceration-MeOH in positive mode; Figure S6. Total ion chromatogram of *Alstonia boonei* leaves maceration-MeOH in negative mode; Figure S7. Total ion chromatogram of *Alstonia boonei* stem bark infusion in positive mode; Figure S8. Total ion chromatogram of *Alstonia boonei* stem bark infusion in negative mode; Figure S9. Total ion chromatogram of *Alstonia boonei* stem bark maceration-EA in positive mode; Figure S10. Total ion chromatogram of *Alstonia boonei* stem bark maceration-EA in negative mode; Figure S11. Total ion chromatogram of *Alstonia boonei* stem bark maceration-MeOH in positive mode; Figure S12. Total ion chromatogram of *Alstonia boonei* stem bark maceration-MeOH in negative mode
